# Learning to rank Higgs boson candidates

**DOI:** 10.1038/s41598-022-10383-w

**Published:** 2022-07-30

**Authors:** Marius Köppel, Alexander Segner, Martin Wagener, Lukas Pensel, Andreas Karwath, Christian Schmitt, Stefan Kramer

**Affiliations:** 1 Johannes Gutenberg University, Mainz, Germany; 2 ETH, Zurich, Switzerland; 3 University of Birmingham, Birmingham, UK

**Keywords:** Experimental particle physics, Computer science

## Abstract

In the extensive search for new physics, the precise measurement of the Higgs boson continues to play an important role. To this end, machine learning techniques have been recently applied to processes like the Higgs production via vector-boson fusion. In this paper, we propose to use algorithms for learning to rank, i.e., to rank events into a sorting order, first signal, then background, instead of algorithms for the classification into two classes, for this task. The fact that training is then performed on pairwise comparisons of signal and background events can effectively increase the amount of training data due to the quadratic number of possible combinations. This makes it robust to unbalanced data set scenarios and can improve the overall performance compared to pointwise models like the state-of-the-art boosted decision tree approach. In this work we compare our pairwise neural network algorithm, which is a combination of a convolutional neural network and the DirectRanker, with convolutional neural networks, multilayer perceptrons or boosted decision trees, which are commonly used algorithms in multiple Higgs production channels. Furthermore, we use so-called transfer learning techniques to improve overall performance on different data types.

## Introduction

In the summer of 2012, a possible candidate for the Higgs boson was discovered by the ATLAS^1^ and CMS^2^ experiments at the Large Hadron Collider (LHC) at the European Organization for Nuclear Research (CERN)^3^. Assuming the observed particle is the Higgs boson predicted by the Standard Model of particle physics (SM), it would complete the SM to a self-consistent theory. The SM is currently the best description for physics at subatomic scales, and it explains most particle physics experiments of the past century. However, a few experimental observations are unexplained by the SM, e.g. the existence of dark matter in our universe^4^. An ongoing effort is therefore to establish limitations of the SM by investigating all of its parts thoroughly. One approach is the precise measurement of properties of the newly discovered Higgs boson, for example through the measurements of Higgs production events via vector boson fusion (VBF)^5^. Since this production channel is overwhelmingly dominated by background events, finding efficient ways to separate them from signal events is crucial.

Since the measured data in the experiment are not labeled and different processes can look very similar in the detector data, it is crucial to find criteria for their distinction on simulated data, for which the corresponding process is known. Typically, this is achieved by first simulating the physical process via Monte-Carlo generators^6–8^. In practice, one only works on these simulated data in order to find ways to classify the process of interest from the detector data. The data measured in the detector follows distinct, overlapping distributions for different physical processes. This leads to the problem that single measurements of different processes can be identical, such that they cannot be distinguished. In order to maximize the certainty with which the processes are classified, machine learning methods are trained on simulated data to classify signal and background events, i.e. events corresponding to the process of interest (in our case the Higgs Boson production via VBF), and those which have similar signature (for example $t{\bar{t}}$ in our case)^9,10^. After training these machine learning methods on simulated data, these models can then be applied to experimentally acquired data and simulated data. In order to analyze the experimental data, the number of measured processes, classified as signal, is compared to the expected number from the simulations. In such experiments it is common practice to only consider the simulated data to fix the model. Only when every part of classification chain is settled the methods are applied to blinded experimentally acquired data. This procedure reduces biased selection of data and p-hacking and has the further advantage that on simulated data it is known which event belongs to which process. More information on the statistical methods applied in high energy particle physics can be found in the paper by Cranmer^11^. A crucial part in this analysis workflow is to develop specialized algorithms that are well-suited for the data at hand.

As the number of measured data points corresponding to an event including a Higgs boson (a signal event) is extremely small compared to background events, learning to rank techniques can be applied to increase the statistical variation of a data set. This is done by using pairs of signal and background events during training^12^, which effectively increases the number of training instances quadratically by using all possible combinations of events. In general, models that address the learning to rank problem sort a list of *n* documents (in the following we use the terms documents and instances synonymously) by their relevance with respect to some query. These models can be separated into three categories according to whether the objective function is computed by considering one, two or a whole list of documents during training. The first approach is called pointwise and is analogous to classifying each document^13–15^ in the sense that a score is predicted on each query-document pair, indicating the relevance of that specific document according to the query. In the pairwise approach, the model attempts to learn the more relevant document out of a pair of two for a given query^16,17^. The last approach is called listwise, where a whole list is used to compute the cost during training^18,19^. It is possible to extend many classification algorithms to the ranking problem, such as decision trees^17^, support vector machines^20^, artificial neural networks^18^ and ensemble boosting^21^. More recently it has been shown that the DirectRanker, a generalization^12^ of the pairwise learning approach RankNet^16^, outperforms several state-of-the-art methods on listwise metrics while needing substantially less training time on pure ranking tasks. This learning algorithm has been proven to be able to learn a total quasiorder on a broad variety of feature spaces by employing a Siamese structured network architecture that inherently guarantees such an order. The model has been used in several different contexts such as learning fair representations on biased data sets^22,23^, for detection of humor in natural language^24^ or ranking structured objects^25^.

In current publications about Higgs boson measurements, boosted decision trees (BDT)^17,26^ or multilayer perceptrons (MLP) are typically used to separate signal and background events^9,10^. Specifically in the VBF channel, current publications make use of deep neural networks^27^ and convolutional neural networks (CNNs)^28^. We show that by using CNNs in combination with the pairwise ranking method DirectRanker, we can outperform current state-of-the-art methods in this field considerably.

Another important aspect to consider is the computational cost of the training data generation. While a full simulation of the interactions of particles with the detector is computationally expensive, it is possible to perform cheaper, simplified simulations which approximate the full interactions. We therefore investigate transfer learning techniques^29–31^, which allows pre-training of models using approximate larger data sets that are easily generated and subsequently retraining them on smaller, more precise data sets. We emulate this here by using data sets that are generated with slightly different parameters.

In summary, the contributions of this paper are as follows:

i. We show that the DirectRanker can be used for the separation of Higgs and background events within the vector boson fusion channel. Moreover, the DirectRanker can be used in combination with convolutional neural networks with significant improvements over state-of-the-art methods.
ii. We perform experiments evaluating the model performance on different signal-to-background ratios and perform comparisons to state-of-the-art machine learning models to illustrate the benefits and use cases of our approach.
iii. We investigate how the performance of numerous models depends on their complexities.
iv. We show that pre-training a convolutional neural network and retraining it on data with a slightly different distribution can increase the overall performance when combining convolutional neural networks and the DirectRanker approache.This technique could be used to pretrain on data that are cheap to generate and decrease computation time in data generation and training and increase overall performance.

## Results

This section covers the results of the performed experiments. Subsequently, the benefits of our approach compared to state-of-the-art algorithms on different signal-to-background ratios and on different model complexities are shown. We also show the results of a retraining approach with the goal of improving the performance on a complete and realistic detector simulation. Finally, a grid search is performed on different data sets to quantify the overall model performance.

### Data set and model complexity analysis results

**Figure 1:**
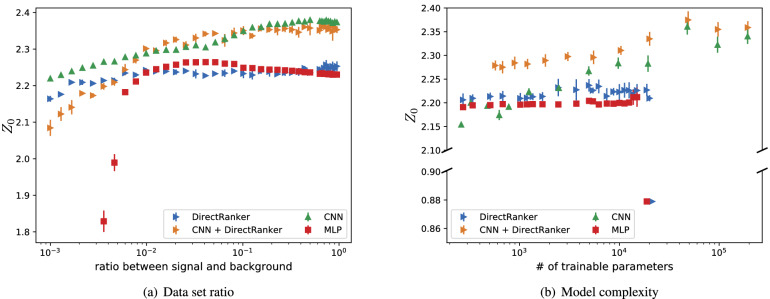
Performance of different machine learning models evaluated using the $Z_0$ metric in a fivefold cross-validation. In (**a**), the models are trained on different signal and background ratios and tested on an equal number of signal and background events. An overview of the generated data set can be found in Table 2 in the experimental setup. In (**b**), the model complexity is increased while using the balanced data set for training and testing. We tested a convolutional neural network (CNN), a multilayer perceptron (MLP), the DirectRanker, and its combination with convolutional layers (CNN + DirectRanker).

In Fig. 1a, one can see that the performance of the CNN increases constantly from low signal to background ratios up to an equal amount of signal and background. The CNN + DirectRanker approach works better than the CNN if the ratio is between $10^{-2}$ and $10^{-1}$, while outside that range the CNN gives better results. For the DirectRanker, however, one observes a constant performance across the whole ratio spectrum with the possible exception of the smallest signal-to-background ratios. The MLP has the same performance as the DirectRanker, but the performance drops if the ratio of signal and background is too low. Overall, the DirectRanker and the MLP have a lower performance than the CNN + DirectRanker and CNN.

The CNN + DirectRanker used in this experiment had a higher minimal complexity than the other models, which makes it hard to train on small amounts of data. Since the DirectRanker has only a low complexity and makes better use of the statistics through pair building, it still performs well on small signal to background ratios, since the possibility of overfitting is lower than for more complex models. Nevertheless, looking at the results for the complexity analysis, one can lower the complexity and still perform well and reduce computational overhead with all models. In Fig. 1b, these results are shown. Note that the complexity of the CNN + DirectRanker model cannot be reduced further than is shown in the plot. The CNN + DirectRanker always performs better than the other models for a given complexity. As for the other models, we observe that the performance of the CNN increases with the model complexity, while the DirectRanker and the MLP display a consistent performance for a wide range of model complexities. However, their performance drops suddenly after they reached a certain complexity. This behavior could be explained by overfitting the training data.

Finding the “sweet spot” of model complexity and signal-to-background ratio is crucial to perform well. Our results suggest that the CNN and the CNN + DirectRanker are suitable candidates for improving vector boson fusion searches.

### Transfer learning results

Table 1 is showing the retraining results for the different data sets which are described in detail in the model section. The results indicate that the retrained model outperforms the CNN + DirectRanker while looking at the data with the changed magnetic field (ATLAS-2.1T). Having the CMS detector or WW2j/Z2j events as precise data sets, the retrained model performs similarly to the CNN + DirectRanker. Overall, this approach shows that the performance of a model trained on precise data can be improved by first training on cheap imprecise data and then retraining the model on precise data. Furthermore, one can save training time since, once the model is pre-trained, it only needs to be fed with precise data, which can be of various kind.

**Table 1:** Results of the transfer learning experiments.

Precise data sets (KS Values)	CNN	CNN + DirectRanker	Retrained CNN + DirectRanker
CMS detector (0.003)	$2.236 \pm 0.013$ $Z_0$	$2.377 \pm 0.015$ $Z_0$	$2.387 \pm 0.008$ $Z_0$
ATLAS-2.1T (0.018)	$2.288 \pm 0.020$ $Z_0$	$2.348 \pm 0.026$ $Z_0$	$2.357 \pm 0.030$ $Z_0$
WW2j/Z2j background (0.002)	$2.433 \pm 0.023$ $Z_0$	$2.787 \pm 0.033$ $Z_0$	$2.763 \pm 0.025$ $Z_0$

The first column of the table shows the generated precise data sets. By calculating the mean of the Kolmogorov–Smirnov^32^ (KS) test over all 32 features, the similarity of the precise data to imprecisely generated $t\bar{t}$ and Higgs events is shown in brackets. For the precise data sets, we generated $t\bar{t}$ and Higgs events with the CMS detector (CMS detector), $t\bar{t}$ and Higgs events with the ATLAS detector having a 2.1T magnetic field (ATLAS-2.1T) and WW2j/Z2j events with the ATLAS detector. The imprecise generated data uses the normal ATLAS detector. The other two columns show the models we compare to our retrained model. Both models are only trained and cross-validated on the precise data sets. Therefore, we use the CNN and the CNN + DirectRanker model. For our retrained model, we first trained CNN layers on imprecisely generated $t\bar{t}$ and Higgs data and then we retrained the full CNN + DirectRanker model on a subsample of the precise data sets. The results for this is shown in the last column. For all model results we report the $Z_0$ metric.

### Overall model performance results

**Figure 2:**
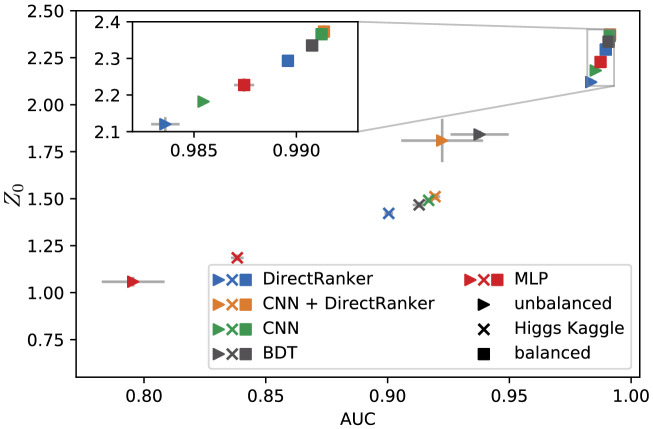
Results of the grid search on $t\bar{t}$ background and Higgs signal data. The performance of the models is measured by $Z_0$ and AUC. The models are trained and tested on different data sets. The models marked with $\vartriangleright$ are trained on unbalanced and tested on balanced data. The ones shown with $\times$ are trained and tested on the Kaggle Higgs boson challenge^33^. The ones plotted with $\Box$ are trained and tested on balanced data.

In Fig. 2, the results of different data sets with $t\bar{t}$ background and Higgs signal data are shown. For the experiments on the Kaggle Higgs boson challenge and on the tests with a balanced train and test data, the CNN + DirectRanker outperforms all other models. For the test with unbalanced training data and balanced test data, the CNN outperforms the other methods. These excellent results consolidate our statement in favor of CNN + DirectRanker and CNN for vector boson fusion searches, since both models are capable of outperforming other state-of-the-art models.

## Discussion

The results presented above show that our approach of using a ranking based machine learning model performs well on simulated ATLAS data for the vector boson fusion channel. The simple DirectRanker model outperforms a MLP in the case of a very unbalanced data sets, see Fig. 1a, with a low number of signal events. This is illustrating one of the strengths of this approach due to the effective increase of training set size by pairwise training. While a pure CNN model outperforms the DirectRanker in almost all cases, our results show that a combinations of the two methods, the CNN+DirectRanker model, can increase overall performance, see Fig. 1a,b. This is especially evident for low complexities, i.e. for a low number of trainable parameters, indicating that with this approach a simpler model can achieve the same performance as a more complex CNN model.

Our results of the overall model performance after conducting a thorough grid search for hyperparameter optimization show that for a balanced data set our CNN+DirectRanker model outperforms all other models tested here, including state-of-the-art methods like BDTs and MLPs. We have used these two methods as a comparison since BDTs are one of the most widely used methods for the classification of high energy physics detector data^9,10^ and MLPs are one of the most promising candidates to further improve performance in this field^27^. It is important to note that we had to re-implement these models and optimized their hyperparameters to compare them to our methods. The relevant publications do not provide the necessary code and documentation to simply reproduce their results and the experimental setups are usually not directly comparable. Furthermore, the data being used in these publications is rarely publicly available. We are therefore convinced, that our method of implementing all models from scratch and performing most experiments on data we generated using commonly used simulation applications^7,8,34,35^, offers the best comparability. Nevertheless, we additionally performed experiments on data from the Kaggle Higgs boson challenge^33^ which quantitatively agrees well with our other experiments.

Another interesting possibility in our CNN+DirectRanker model is illustrated in the results using transfer learning. We show that by pre-training the CNN part on some data and subsequently training the CNN+DirectRanker on data which is from a slightly different distribution we can outperform the not pre-trained model in some cases. This can be interesting in cases where data from one distribution is cheap to generate and data from the other is more expensive to generate, as is often the case in high energy physics data. While the performance gain is not very large, this approach offers a further benefit by possibly reducing training time. Once a pre-trained CNN model is available the full model can be quickly trained again on various kinds of data. It is important to note that our results on this topic do not necessarily show the full potential of this method. The data sets for pre-training are chosen somewhat arbitrarily, experiments on actual more precise detector simulations for the ATLAS experiment would probably be more conclusive. Another interesting use case is the retraining with different backgrounds. In the physical context, this allows to refine the results later into an existing analysis by introducing more background processes which were previously neglected.

Our work shows that the DirectRanker and especially the combination of CNN+DirectRanker is a promising machine learning approach for ranking high energy physics detector data. This is interesting both because of the high performance compared to other state-of-the-art methods and due to the novel network architecture. Our results agree well with the original presentation of the DirectRanker^12^ showing high performance on unbalanced data and low complexity models. This performance furthermore remains stable over a wide range of model complexities. We also exploit the possibility of flexibly expanding this original DirectRanker model with more complex approaches like CNNs as envisioned in the original publication. While these points are some of the main strengths the combination of CNNs and the DirectRanker make the model somewhat more complicated and add further hyperparameter combinations to be adjusted simultaneously. This means that the design of the network architecture becomes more intricate and more care has to be taken in the hyperparameter optimization.

In summary, we see the following strengths and weaknesses in our presented methods:

+: Our model shows high performance compared to state-of-the-art methods independent of the data set size, even if the model complexity is low.
+: By introducing a novel approach (CNN+DirectRanker), we showed that optimizing the model architecture itself might be more worthwhile than increasing the model complexity.
+: Our models enable efficient transfer learning for cheaper data generation.
−: Our transfer learning tests lack results on a more rigorous and expensive detector simulation.
−: The combination of different models introduces an increase of hyperparameters to be optimized.

Further improvements and research are possible in multiple aspects of this work. It might be possible to improve the performance by introducing better feature parts for the DirectRanker. While CNNs have proven to produce competitive results here, it might be possible to find network architectures that exploit the correlations between data features in a more optimized way. It might also be instructive to further investigate our transfer learning approach. One could conduct experiments on different kinds of data sets that better represent the notion of precise and less precise data or try to pre-train different parts of the model. Additionally, it would be interesting to check if our approach also works for data from other physical processes than the Higgs production via VBF. Different production channels of the Higgs boson or altogether different particle physics processes and experiments would be possible candidates here.

## Methods description

In this section, we elaborate the machine learning approaches used in this work in more detail. The two primary methods that have been used are convolutional neural networks (CNNs) and the DirectRanker. To provide a model combining the benefits of these approaches, we will subsequently illustrate how to join these two techniques. Furthermore, we explain in detail the performed experiments we did to evaluate the performance of the different approaches.

### Model descriptions

#### Convolutional neural network

Convolutional neural networks (CNNs)^36^ are robust against scaling, shifting and disordering of the input data by taking local dependencies into account. Classifying local dependencies like a nose in a human face or parts of music or sentences, makes the approach a favourable candidate for image, sound and text analysis. In the same way, they can be employed in classification of the data at hand since some of the adjacent features also exhibit meaningful, physical dependencies. To ensure dependent variables from the same physical origin can be employed correctly, we ensure these variables also exhibit a close proximity within the input data ordering. For example, the features which correspond to the leptons are grouped together in the input representation. The architecture of the used CNN in this paper was mainly taken from the Master thesis of Pensel^28^. In the supplementary material, a more concrete motivation of the used parameters is given. In the following, CNN layers are used to generate additional feature combinations for the ranking model.

#### DirectRanker

**Figure 3:**
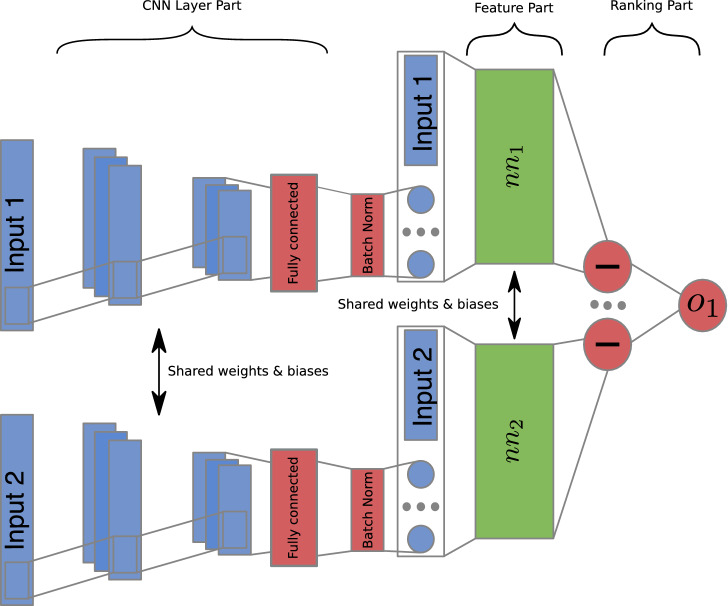
Adaptation of the DirectRanker architecture to use convolutional layers to extract additional features from the inputs. The two convolutional networks share their parameters and architecture. After the convolutional layers, a fully connected layer is used the further reduce the extracted features before they are concatenated with the original ones. The concatenated features are then fed into the original DirectRanker model^12^. During training, a batch normalization layer^37^ is used before the extracted features are added to the primary ones to prevent overfitting.

As a generalization of RankNet^16^, the DirectRanker was introduced by Köppel et al.^12^. A total quasiorder on the feature space is induced, since the model architecture leads to a pairwise ranking function that is reflexive, total and transitive by construction. The authors showed that this method can outperform other state-of-the-art ranking approaches including list-wise approaches while only requiring a fraction of the computation time and smaller amounts of data for training. The structure of the model starts with two identical feature extraction networks, called feature part (see $nn_1$ and $nn_2$ in Fig. 3). Originally these two networks consist out of simple, fully connected neural networks but could in general be more complex functions of the input features. This is how we extend that model here by using CNN layers for further feature combination, as illustrated in the following section. The outputs of the feature extraction part are subsequently subtracted from each other. Finally, the single neuron ($o_1$) takes the output of this subtraction and maps the pair of data instances onto a single number *r*, which represent the ranking function of the model, where $r>(<)\,0$ means the first (second) instance is the preferable one. This part of the DirectRanker is called ranking part. By choosing the two feature extraction networks to be identical and the output neuron to obtain a sign-conserving activation and zero bias, we obtain reflexivity, totality and transitivity of the relation defined by *r* with respect to the two input instances.

#### DirectRanker for ranking Higgs boson candidates

By incorporating CNN layers to the feature extraction part of the DirectRanker, the used approach can be extended to be more suitable for ranking Higgs boson candidates. Specifically, the CNN layers are used to extract new features from the original ones. The combination of original and new features are then used as input to the DirectRanker, as described above and depicted in Fig. 3. The number of neurons in the final layer of the CNN extraction part can be used to fine-tune the influence of the convolutional layers to the overall ranking prediction. Similarly to the simple DirectRanker, both CNN layers employed in the two feature parts have to be identical to ensure the overall properties of the ranking function. To provide features of similar magnitude to the fully connected network, an additional batch normalization layer^37^ is applied to the output of the CNN. The so constructed combination will be called CNN + DirectRanker throughout this work.

### Experimental setup

#### Data generation

The used signal and background processes are simulated using Monte-Carlo techniques. The Higgs signal is generated with MadGraph5^6^ at NLO (next-to-leading order in perturbation theory) using Higgs Effective Field Theory^34,35^ and combined with the parton shower from PYTHIA8^8^. The background processes $t\bar{t}$, *WW*2*j* and *Z*2*j* are simulated using the same generators. For generating the detector response, the DELPHES framework for fast simulation of a generic collider experiment^7^ was employed. Our main focus was set to simulate data for the the ATLAS detector. Before employing the data for the performance experiments, basic quality cuts are performed^38^. The used cuts require having 2 leptons and > 2 jets in the final state. The features extracted from these simulated data contain the energy, the transverse momentum, the polar angle in the plane transverse to the particle beam, and the pseudorapidity for the first, second, and third jet and for the first and second lepton. Pseudorapidity describes the angle of a particle relative to the beam axis. Moreover, the number of jets, the number of leptons, and the missing transverse energy is provided. Out of these features 9 further physically motivated features are constructed. An accurate table of all employed features can be found in Supplementary Table 1 of the supplemental material. Apart from this, the detector parameters used in the DELPHES framework were changed to generate different feature distributions. For this purpose, the detector card from CMS was used and also the magnetic field of the ATLAS detector was changed from 2T to 2.1T. In Table 2 an overview of all the simulated data is shown. Frequently, the same number of signal and background events are simulated to have a balanced data set to train the machine learning model. The models are trained on these numerically generated data and could later be used to classify signal events on data from the experiment. In contrast to the simulated data, the real detector data contains only a minor fraction of signal events, as the cross section of signal events is relatively small compared to the background processes. We experiment with both signal-to-background ratios by splitting the data into two subsets to evaluate the model’s performance when trained on balanced data and on a realistic signal-to-background ratio. One subset contains around 300 k Higgs events and 300 k $t\bar{t}$ events, later called the balanced data set. The other subset contains 300 of Higgs events and 300 k $t\bar{t}$ events, which we refer to as the unbalanced data set. The number of events are calculated with an integrated luminosity of ${156}{\text {fb}^{-1}}$.

**Table 2:** Overview of the number of instances in the generated data sets after applying the quality cut of 2 leptons and > 2 jets.

DELPHES card	# $t\bar{t}$	# WW2j	# Z2j	# Higgs
CMS Detector	400 k	–	–	400 k
ATLAS-2.1T	42 k	–	–	34 k
ATLAS	2 M	280 k	230 k	340 k

ATLAS-2.1T indicates that the magnetic field in the ATLAS DELPHES detector card was changed from 2T to 2.1T.

#### Evaluation metric

For the evaluation of the models the following two metrics are used: Firstly, the general known AUROC (Area Under the Receiver Operating characteristic Curve), referred to here as AUC, is employed. Secondly, the likelihood-based statistical significance ($Z_0$) for the discovery of a new process^39^ is used. We use $Z_0$, defined as

1 $$\begin{aligned} Z_0 = \sqrt{2\left( (s + b)\ln \left( 1 + \frac{s}{b}\right) - s\right) }, \end{aligned}$$

which represents a special case of the approximate median significance (AMS) metric used for the Kaggle Higgs boson challenge^33^. *s* is equal to the number of true positives and *b* to the number of false positives scaled to the number of events to be expected in the detector.

#### Computing infrastructure

All neural network models were built with Tensorflow^40^. The scikit-learn 0.24.2 library^41^ was utilized for boosted decision trees. The Higgs events were generated with MadGraph5^6^ using Higgs Effective Field Theory^34,35^ and combined with the parton shower from PYTHIA8^8^. The background processes $t\bar{t}$, *WW*2*j* and *Z*2*j* are simulated with MadGraph5 and the parton shower from PYTHIA8 only. The detector response is generated with the DELPHES framework for fast simulation of a generic collider experiment^7^. All experiments and simulations were executed on an Arch Linux system with an Intel^®^ Core™i7-6850K CPU @ 3.60GHz with 32 GB of RAM and an nVidia GeForce GTX 1080 Ti with 10 GB of memory. In Table 3 the training time of the different models evaluated over fivefolds of the balanced data set is given. The used hyperparameters are those of the best performing models (see the Supplement).

**Table 3:** Mean training time of the different models evaluated on fivefolds of the balanced data set.

Model	Runtime [s]
DirectRanker	$81 \pm 2$
CNN+DirectRanker	$162 \pm 8$
CNN	$16 \pm 0$
MLP	$60 \pm 4$
BDT	$789 \pm 64$

#### Setup data set and model complexity analysis results

To evaluate the model performance of different neural network architectures, we compared the DirectRanker, a convolutional neural network (CNN), a multilayer perceptron (MLP) and the combination of the DirectRanker with convolutional layers (CNN + DirectRanker). The data employed in the following two experiments uses $t\bar{t}$ for background and Higgs for signal events. Both data sets are simulated with the ATLAS detector card using DELPHES.

For the first experiment, we evaluated the model performance while changing the data set ratio of the training data. For this, we increased the ratio between signal and background samples in the training data set, starting from a minor fraction of signal events up to an identical number of signal and background events. For testing, we always used an equal number of signal and background events. The DirectRanker was set up with two hidden layers in the feature part, where the first one contained 20 neurons and the second one only 2. For the MLP, we fixed the architecture to three hidden layers, where the first one had 64, the second 20, and the last one 2 neurons. Beside this, we employed dropout layers after each hidden layer in the the MLP approach. We additionally used weight regularization to prevent the model from overfitting. The CNN model had a kernel size of 3 with 64 filters in one convolutional layer followed by a fully connected layer with 50 neurons. For the CNN + DirectRanker model, we used the parameter setting and architectures as for the DirectRanker and the CNN individually. The results are shown in Fig. 1a, where the mean value of $Z_0$ of a fivefold cross-validation is used as performance metric.

Figure 1b presents the results when the ratio between signal and background is balanced in both, the training and the test data sets. In this test, the model complexity is changed to evaluate the relation between model performance and model complexity. The complexity of the model is expressed as the absolute number of trainable parameters. To increase the number of trainable parameters, we increased the number of layers for the DirectRanker and the MLP. More specifically, we selected the number of neurons in each layer to be $n+m+2$, where *n* is the number of input neurons (32) and *m* the number of output neurons (1) following the approach of Deep Narrow Networks^42^ to fulfill the requirements of the Universal approximation theorem. The CNN contained only one convolutional layer, a kernel size of 3 and one fully connected layer. For changing the complexity, we increased the number of filters in the convolutional layer from 2 to 128 filters and varied the number of neurons in the fully connected layer from 2 to 50. The CNN + DirectRanker used the same parameters as the CNN, while the two ranking parts had both one hidden layer with 10 neurons. Figure 1b reports the results for each model at different complexities using a fivefold cross-validation with $Z_0$ as performance metric.

#### Setup transfer learning results

A realistic detector simulation can be computationally extremely expensive. Therefore, only a few events per channel can be generated. Using transfer learning techniques can result in comparable or even better results as for precisely generated data (we use the term imprecise and precise as synonyms for fast and full detector simulation, respectively). As the precise simulations are exclusively available to the ATLAS Collaboration, we instead emulated the precise data by slightly tweaking the simulation parameter in order to generate data from a different, yet similar, distribution. We introduced three distinct emulations of the precise data which are detailed in the section on data generation. In the following we refer to these emulations as precise data in the context of our experiments. The data generated with the standard ATLAS Delphes-card are referred to as imprecise. This allowed us to test the transfer learning approach with a CNN model that was trained on the imprecise data and then extended with the ranking part of the DirectRanker for transfer training on the precise data. The ranking model constructed in this way can converge faster on the precise data, since the pre-trained CNN layers provide a much better adapted starting point than randomly assigned weights^29^. We compared this approach to a CNN + DirectRanker model, which was trained on the whole data set without any pretraining, and to a CNN model, which was pre-trained on the same imprecisely generated data, and then also retrained on the precise one. To quantify the difference of the data sets and their underlying feature distributions, we used the Kolmogorov–Smirnov^32^ (KS) test. The results for individual features are summed up individually and normalized. To determine the best performing pre-trained CNN model, a fivefold cross-validation was performed using 150,000 imprecise signal and background events each. Subsequently, the best performing CNN model was selected and the ranking component was retrained on 30000 samples of precise data. The hyperparameter optimization for the retrained model was done by splitting the precise data into a fivefold split. On each fold, an internal fivefold cross-validation was conducted to find the best hyperparameters, while model performance was evaluated on the external folds. The retraining of the CNN-only approach was performed employing a similar procedure. As for the CNN + DirectRanker, the imprecise data for the CNN model and the precise data for the retrained model were combined to perform a hyperparameter optimization. The model performance was again evaluated using the same separate split of precise data. In Table 1, the mean of $Z_0$ from the external fivefold cross-validation and the KS values are shown.

#### Setup overall model performance

To assess overall performance, we compared our model on different data sets with current state-of-the-art methods. Beside the different neural network approaches, the BDT, which represent the state-of-the-art method for the vector boson fusion channel, was included in this evaluation. For these, three data sets are used. The first one included a balanced number of signal and background events in the training and test set. The second one provided an unbalanced number of signal and background events in the training set but a balanced amount in the test set. The last data set in this experiment is the Higgs Boson Challenge^33^. For all model-data-set-combinations, we performed an internal fivefold grid search over different hyperparameters. The model performance is evaluated using AUC and $Z_0$ on an external fivefold split. The best overall hyperparameter values were evaluated by counting how many times they performed the best on the external 5 sub-folds. Some hyperparameters produced no convincing winner, since there was no majority in the overall performance.

## Supplementary Information

Supplementary Information.
