# Supplementary material for: Learning to rank Higgs boson candidates
Source: Sci Rep. 2022 Jul 30;12:13094. doi: 10.1038/s41598-022-10383-w (PMC9338962; doi:10.1038/s41598-022-10383-w)
Supplement: Supplementary file 1 — Supplementary Information. [file 41598_2022_10383_MOESM1_ESM.pdf]

Supplementary Material

DirectRanker

Supplementary Table 1. Overview of the hyperparameters used for training the DirectRanker. The best overall performing points are indicated in boldface. Dropout and L2 weight regularization are dependent on the size of the network.

Parameter	Values
activation nn	**tanh**, relu
activation o_1	**tanh**, linear
# layers	2..8
# neurons per layer	5..256
dropout	0%, 50%
L_2 weight regularization	0.0, 0.0001
cost function	L_2, cross-entropy
batch size	50, 100, **200**, 500
learning rate	**0.001**
learning rate decay rate	**1**
learning rate decay steps	**1000**
optimizer	**Adam**, Nadam, SGD
epoch	**10**, 50, 100
validation size	**0.1**
early stopping look-back	**3**, 6, no

The DirectRanker is a pairwise ranking approach. Pairwise ranking algorithms require two instances and decide which of them is more relevant. To achieve a consistent ranking, the DirectRanker implements a total quasiorder $\succeq$ on the feature space $\mathscr{F}$ through its ranking function $r : \mathscr{F} \times \mathscr{F} \to \mathbb{R}$ with $x \succeq y \iff r(x,y) \geq 0$. This function satisfies the following properties:

- Reflexivity: $r(x,x) = 0$
- Antisymmetry: $r(x,y) = -r(y,x)$
- Transitivity: $(r(x,y) \geq 0 \wedge r(y,z) \geq 0) \Rightarrow r(x,z) \geq 0$

To implement such a function, the model is divided into two parts. The first part is called feature part, made of fully connected layers. It learns a deep representation of the input instances. In Figure 3, this part is built out of two subnetworks nn_1 and nn_2 that share their parameters. As long as the two parts yield the same output for the same input, they can be made of any kind of layers. The extracted representation of the two subnetworks nn_1 and nn_2 is first substracted and than fed into the ranking part of the DirectRanker. This second part of the model is made of one neuron with a sign conserving activation and no bias. In Figure 3, this part is marked with o_1. Fulfilling these conditions, the authors have proven that the DirectRanker[1] is able to implement a total quasiorder.

To evaluate the best hyperparameters, the authors of the DirectRanker explored multiple model architectures on common benchmark ranking tasks. For the experiments in this work, we extended the explored parameter space. All used parameters are shown in Table 1, where the overall best are indicated in boldface. As observed in the original paper[1], we also found that larger models perform better with dropout or weight regularization, while a smaller net holds the same performance with the overall training time being shorter. Since the original DirectRanker was implemented in Tensorflow v1.15.0, we re-implemented it in Tensorflow v2.5.0[2]. This implementation allowed us to train the model in epochs using the whole data set multiple times, while the initial version was trained in one epoch only. We also implemented the possibility to use early stopping using a look-back parameter and stop the training if the performance does not increase on a separate validation set. Beside the L_2 cost function, we evaluated the performance using a cross-entropy cost. Furthermore, we explored Nadam[3], an Adam-Optimizer using Nesterov Momentum and stochastic gradient descent (SGD)[4] additionally to the standard Adam-Optimizer[5] used in original work. Both of these changes lead to no significant increase in performance.

Convolutional Neural Network

The motivation why convolutional neural networks (CNN)[6] should work well for high-energy physics analysis is that they are to some degree invariant to shifting, scaling and distortion of the input data. Typically this is effective for image, sound or text

Supplementary Table 2. Overview of the hyperparameters used for training the convolutional network. The best overall performance is indicated in boldface. The permutation layer represents a fully connected layer with the identical number of neurons as the input size. The task of this layer is to reorder the input features before they are fed into the convolutional layers.

Parameter	Values
convolutional activation	**SRelu**, Relu
dense activation	**Relu**, tanh
permutation layer	**false**, true
# convolutional layers	1..6
# convolutional filters	16..128
# kernel	2, **3**
# dense layers	1..3
# neurons per dense layer	25, 50, 100, 150
locally connected	**true**, false
L_2 weight regularization	0.0, 0.0001
cost function	**cross-entropy**
batch size	50, 100, **200**, 500
learning rate	**0.001**
learning rate decay rate	**1**
learning rate decay steps	**1000**
optimizer	**Adam**, Nadam, SGD
epoch	**10**, 50, 100
validation size	**0.1**
early stopping look-back	**3**, 6, no

analysis, where the order of the data contains some information. Since the data collected in high-energy physics experiments is similar to image data, the properties of the CNNs can be beneficial. For many analyses in high-energy physics experiments, however, reconstructed data is used, where the given image-like data is converted into actual properties of physical particles. Although this can be the case, properties of the same particle are frequently placed next to each other in the reconstructed data. Therefore, numerous properties of one particle can be combined by using a CNN, which bears some potential to improve the classification result in this way. In this work, we always reconstruct 3 particles or jets having values, like transverse momentum or energy, located next to each other in the data. This motivated to use CNNs with 3 kernels able to combine the 3 values of the transverse momentum, and so forth.

In Table 2, the used hyperparameters evaluated for the numerous CNN models used in this work are shown. CNNs are typically composed of multiple convolutional layers having different filter sizes. On top of these convolutional layers, fully connected layers are stacked to further process the extracted information from the convolutional layers. For the convolutional layers, the best values was having one to two layers with 64 filters. The best activation function used for the convolutional layer was an S-shaped Rectified Linear Unit (SRelu)[7], which is able to learn both convex and non-convex functions. The best performing hyperparameters for the fully connected layers were 50 neurons and using a Relu function. If the number of layers is greater, weight regularization performed better, while for fewer layers no weight regularization was chosen. The used cost function for this model was cross-entropy. The best batch size was 200 with 10 epochs having the same parameters as the DirectRanker. In addition, the Adam-Optimizer was employed and the early stopping look-back was set to 3.

Combination of Ranking and CNN Layers

For combining the benefits of the CNN and the DirectRanker, we propose a combination of both. We use CNN layers to extract feature combinations, which are then added to the original features, before they are fed into the DirectRanker. Figure 3 shows this combination. To make this combination work, a couple of difficulties have to be overcome. First of all, the two networks in the ranking part of the DirectRanker need to be identical. To fulfil this, the CNN layers and the subsequent fully connected layer needs to have shared weights and biases. Another problem occurs when combining the extracted features with the original ones, since the extraction will likely transform the features such that they obtain a different distribution than the original ones. Therefore, a batch normalization layer[8] was inserted for normalizing the extracted features. In addition, the number of extracted features is important: It determines which of the two approaches has more influence on the result. If the last layer of the CNN containts a substantial number of neurons, the overall results depends more on the CNN rather than on

Supplementary Table 3. Overview of the hyperparameters used for training the combination of ranking and CNN layers. The best overall performance is indicated in boldface. The permutation layer represents a fully connected layer with the same number of neurons as the input size. The task of this layer is to reorder the input features before they are fed into the convolutional layers.

Parameter	Values
convolutional activation	**SRelu**, Relu
cnn dense activation	**Relu**, tanh
permutation layer	**false**, true
# convolutional layers	1..6
# convolutional filters	16..128
# kernel	2, **3**
# dense layers	1..3
# neurons per dense layer	25, 50, 100, 150
batch normalization	**LayerNormalization**, RandomBatchNorm, false
concatenation size	**6**, 3, 10
activation *nn*	**tanh**, relu
activation o_1	**tanh**, linear
# layers *nn*	2..8
# neurons per layer *nn*	5..256
dropout *nn*	0%, 50%
L_2 weight regularization	0.0, 0.0001
cost function	L_2, cross-entropy
batch size	50, 100, **200**, 500
learning rate	**0.001**
learning rate decay rate	**1**
learning rate decay steps	**1000**
optimizer	**Adam**, Nadam, SGD
epoch	**10**, 50, 100
validation size	**0.1**
early stopping look-back	**3**, 6, no

the DirectRanker part. In our hyperparameter optimization, we found that having this number moderate to low gives the best results. Training the combination can be done in the same way as the DirectRanker is trained. This leads to the advantage that the number of instances presented to the network increases, since it can sample from all combinations between signal and background. This is in contrast to standard classification, which samples just from either signal or background.

In Table 3, all hyperparameters of the model are shown. The bold ones obtained the most outstanding performance, while regularization parameters like dropout or weight regularization worked better for bigger networks. On average, the hyperparameters finally taken for the combination are more or less equal to the best ones for the individual models. For the normalization layer, the best hyperparameter was layer normalization[9]. In contrast to batch normalization, the mean and variance during layer normalization is calculated from the summed inputs of a layer on a particular training case.

Multilayer Perceptron

The used multilayer perceptron (MLP) maintained an input layer with the identical size as the input data, various numbers of hidden layers, and one output layer containing one neuron. The hyperparameters used for the MLP can be seen in Table 4. The size of the fully connected hidden layers was changed from 2 to 8 layers containing 5 to 256 neurons per layer. The number of layers was always set in a descending order. For the activation function for the hidden layers and the output layer, tanh and Relu were used, while tanh performed best in most of the experiments. Once more we saw that bigger networks work better when dropout or L_2 weight regularization was applied. Moreover, smaller ones did not need such regularization, while still achieving similar performance. The best batch size, epoch numbers, and early stopping look-back were once again: 200, 10, and 3. In addition, the best optimizer was Adam.

Supplementary Table 4. Overview of the hyperparameters used for training the multilayer perceptron. The best overall performance is indicated in boldface.

Parameter	Values
# layers	2..8
# neurons per layer	5..256
activation	**tanh**, relu
L_2 weight regularization	0.0, 0.0001
dropout	0%, 50%
batch size	50, 100, **200**, 500
learning rate	**0.001**
learning rate decay rate	**1**
learning rate decay steps	**1000**
optimizer	**Adam**, Nadam, SGD
epoch	**10**, 50, 100
validation size	**0.1**
early stopping look-back	**3**, 6, no

Supplementary Table 5. Overview of the hyperparameters used for training the boosted decision tree. The best overall performance is indicated in boldface.

Parameter	Values
# estimators	50, 100, **150**
learning rate	**0.25**, 0.5, 0.75
max tree depth	1, **2**, 4
min # samples per leaf	**10**, 20, 30

Boosted Decision Tree

The current state-of-the-art algorithm used for high-energy physics search is the boosted decision tree. The implementation evaluated in this work is from the scikit-learn 0.24.2 library[10]. The AdaBoost algorithm[11] is used to boost a standard decision, including a maximum depth and a maximum coverage hyperparameter.

The explored hyperparameters for this method are shown in Table 5. The best value for the underlying decision tree was obtained by a maximum tree depth of 2, while having a minimum number of samples per leaf of 10. For the AdaBoost algorithm, the best learning rate remains 0.25, while employing 150 estimators.

Feature Table

Supplementary Table 6. Features of the used data for the fast detector simulation with ATLAS as detector card.

Feature	description
jetPt0	
jetPt1	
jetPt2	
jetEta0	
jetEta1	
jetEta2	
jetPhi0	
jetPhi1	
jetPhi2	
jetE0	
jetE1	
jetE2	
nJet	
nbJet	jet0->BTag + jet1->BTag + jet2->BTag
DYjj	0.5 * log((jetE0 + jetPt0) / (jetE0 - jetPt0)) - 0.5 * log((jetE1 + jetPt1) / (jetE1 - jetPt1))
Mjj	(jet0->P4() + jet1->P4()).M()
lepPt0	
lepPt1	
lepEta0	
lepEta1	
lepPhi0	
lepPhi1	
lepE0	
lepE1	
MET	
nLeps	
DPhill	min(abs(phi1-phi2), abs(2.*PI-abs(phi1-phi2)))
Mll	mu0/e0->P4() + mu1/e1->P4()).M()
MT	sqrt((lepE0 + lepE1 + MET)*(lepE0 + lepE1 + MET) - (lepPt0 + lepPt1 + MET)*(lepPt0 + lepPt1 + MET))
PtTot	MET + lepPt0 + lepPt1 + jetPt0 + jetPt1 + jetPt2
SumOFMLepxJety	SumOFMLepxJety_11 + SumOFMLepxJety_12 + SumOFMLepxJety_21 + SumOFMLepxJety_22
contOLV	abs(lepEta0 - sumEta) / dEta + abs(lepEta1 - sumEta) / dEta

References

1. Köppel, M. *et al.* Pairwise learning to rank by neural networks revisited: Reconstruction, theoretical analysis and practical performance. In *Machine Learning and Knowledge Discovery in Databases*, 237–252 (2020).
2. Abadi, M. *et al.* TensorFlow: Large-scale machine learning on heterogeneous systems (2015). Software available from tensorflow.org.
3. Dozat, T. Incorporating nesterov momentum into adam (2016).
4. Ruder, S. An overview of gradient descent optimization algorithms. *arXiv preprint arXiv:1609.04747* (2016).
5. Kingma, D. P. & Ba, J. Adam: A method for stochastic optimization (2017). 1412.6980.
6. Lecun, Y., Bottou, L., Bengio, Y. & Haffner, P. Gradient-based learning applied to document recognition. *Proc. IEEE* **86**, 2278–2324 (1998).
7. Jin, X. *et al.* Deep learning with s-shaped rectified linear activation units (2015). 1512.07030.
8. Ioffe, S. & Szegedy, C. Batch normalization: Accelerating deep network training by reducing internal covariate shift. *CoRR* **abs/1502.03167** (2015). 1502.03167.
9. Ba, J. L., Kiros, J. R. & Hinton, G. E. Layer normalization (2016). 1607.06450.
10. Pedregosa, F. *et al.* Scikit-learn: Machine learning in Python. *J. Mach. Learn. Res.* **12**, 2825–2830 (2011).

11. Freund, Y. & Schapire, R. E. A desicion-theoretic generalization of on-line learning and an application to boosting. In *European conference on computational learning theory*, 23–37 (Springer, 1995).
